# Triterpene Esters: Natural Products from *Dorstenia arifolia* (Moraceae)

**DOI:** 10.3390/molecules18044247

**Published:** 2013-04-11

**Authors:** Catharina E. Fingolo, Thabata de S. Santos, Marcelo D. M. Vianna Filho, Maria Auxiliadora C. Kaplan

**Affiliations:** 1 Núcleo de Pesquisas de Produtos Naturais, Universidade Federal do Rio de Janeiro, Av. Carlos Chagas Filho, 373, Centro de Ciências da Saúde, Bloco H, 1º andar, sala 06, Cidade Universitária, CEP: 21941-902, Rio de Janeiro, RJ, Brazil; E-Mails: thata_ssantos@yahoo.com.br (T.S.S.); makaplan@uol.com.br (M.A.C.K.); 2 Instituto de Pesquisas Jardim Botânico do Rio de Janeiro, Unidade de Botânica Sistemática, Rua Pacheco 915/sala 208, Rio de Janeiro-RJ/Jardim Botânico, CEP: 22460-030, Rio de Janeiro, RJ, Brazil; E-Mail: marceloviannafilho@gmail.com

**Keywords:** *Dorstenia arifolia*, Moraceae, triterpenes, natural product chemistry

## Abstract

The phytochemical study of *Dorstenia arifolia* Lam. (Moraceae) has led to the identification of 18 triterpenes esterified by fatty acids, five triterpenes without esterification, 12 triterpenes esterified by acetic acid, together with a known furanocoumarin: α-amyrin (**1**), β-amyrin (**2**) α-amyrin acetate (**3**) β-amyrin acetate (**4**), α-amyrin octanoate (**5**), β-amyrin octanoate (**6**), α-amyrin decanoate (**7**), β-amyrin decanoate (**8**), α-amyrin dodecanoate (**9**), β-amyrin dodecanoate (**10**), α-amyrin tetradecanoate (**11**), β-amyrin tetradecanoate (**12**), α-amyrin hexadecanoate (**13**), β-amyrin hexadecanoate (**14**), glutinol (**15**), glutinyl acetate (**16**), 11-oxo-α-amyrin (**17**), 11-oxo-β-amyrin (**18**), 11-oxo-α-amyrin acetate (**19**), 11-oxo-β-amyrin acetate (**20**) 11-oxo-α-amyrin octanoate (**21**) 11-oxo-β-amyrin octanoate (**22**), 11-oxo-α-amyrin decanoate (**23**), 11-oxo-β-amyrin decanoate (**24**) 11-oxo-α-amyrin dodecanoate (**25**) 11-oxo-β-amyrin dodecanoate (**26**), ursa-9(11),12-dien-3-yl acetate (**27**), oleana-9(11),12-dien-3-yl acetate (**28**), ursa-9(11),12-dien-3-yl decanoate (**29**), oleana-9(11),12-dien-3-yl decanoate (**30**), 12,13-epoxyolean-3-yl acetate (**31**), 12,13-epoxyolean-9(11)en-3-yl acetate (**32**), taraxeryl acetate (**33**), lupenyl acetate (**34**), lanosta-8,24-dien-3-yl acetate (**35**) and psoralen (**36**). The identification of the triterpene compounds isolated as isomeric mixtures obtained from the hexane extract was based mainly in mass spectra and ^13^C-NMR data. The long-chain alkanoic acid esters of the triterpenes α- and β-amyrin; 11-oxo-α- and 11-oxo-β-amyrin; ursa- and olean-9(11),12-dien-3-yl; have not been reported before in the literature as constituents of the *Dorstenia* genus.

## 1. Introduction

The genus *Dorstenia* (Moraceae) is a large genus occurring in the tropics around the World that encompasses 170 herbaceous perennials species with succulent rhizomes [1,2,3,4,5,6,7]. This genus is recognized as a rich source of prenyl and geranyl-substituted coumarins, chalcones, flavanones, flavones, flavonols [7,8] and terpenoids.

Triterpenes are a class of natural products found especially in plants. The triterpene acids exhibit important biological and pharmacological activities, including anti-inflammatory, antimicrobial, antiviral, cytotoxic and cardiovascular effects [9]. The compounds α-amyrin and β-amyrin, commonly found in medicinal plants, have many bio-active properties. Some studies have demonstrated that the α/β amyrin triterpene mixture also has many biological functions, including analgesic, antimicrobial, anti-inflammatory properties [10].

Some *Dorstenia* species show the strong ethnobotanical indications concerning anti-snake bite poisoning properties. Such effects may be related to the presence of triterpenoids [11]. The presence of triterpenes esterified by fatty acids has been a common characteristic in plant species from Brazilian Restinga [12] mainly in the Erythroxylaceae [13].

In Brazil, pharmacological information about the *Dorstenia* genus are very few [14]. No previous phytochemical study on *Dorstenia arifolia* Lam. has been reported. This paper deals with the isolation and the structural elucidation of 18 long-chain alkanoic acid esters of some triterpene skeletons, five triterpenes, 12 triterpenes esterified by acetic acid and only one already known furanocoumarin. The present study has focused on the analysis of terpenoidal compounds from *Dorstenia arifolia*, using phytochemical methodology. This study may be an excellent tool to show the value of classical phytochemical analysis procedures based on chromatographic isolation combined with spectroscopic identification, for the analysis of low-polarity plant extracts [11].

## 2. Results and Discussion

Powdered leaves and rhizomes of *D. arifolia* were successively extracted with *n*-hexane. The extracts were submitted to repeated column chromatography to afford various pentacyclic triterpenes esterified by fatty acid and a coumarin. The ^1^H- and ^13^C-NMR as well as the MS of the isolated compounds were consistent with the literature records.

**Figure 1:**
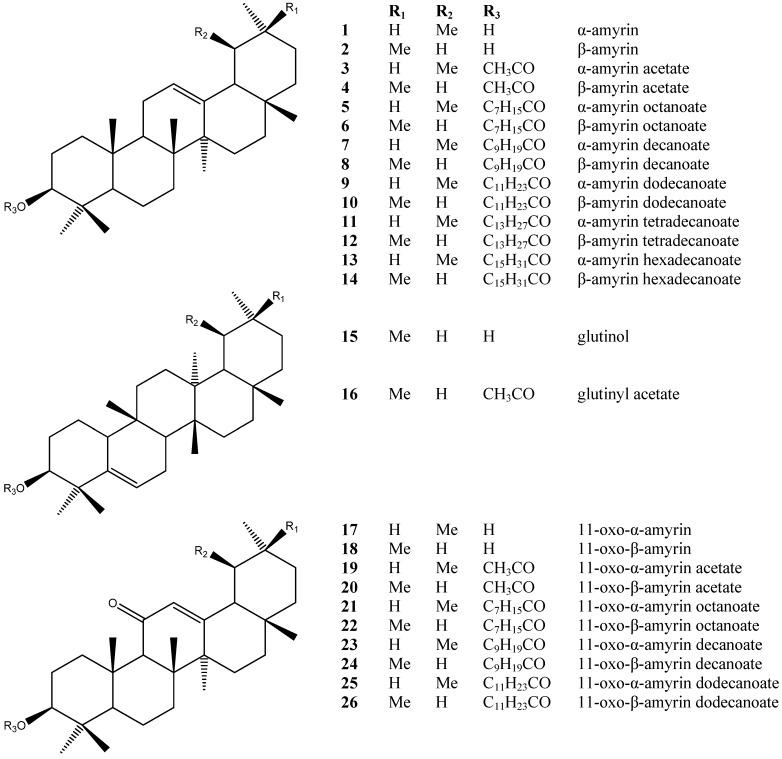
Chemical structures of the compounds detected by GC-MS in *Dorstenia* *arifolia*.

**Figure 2:**
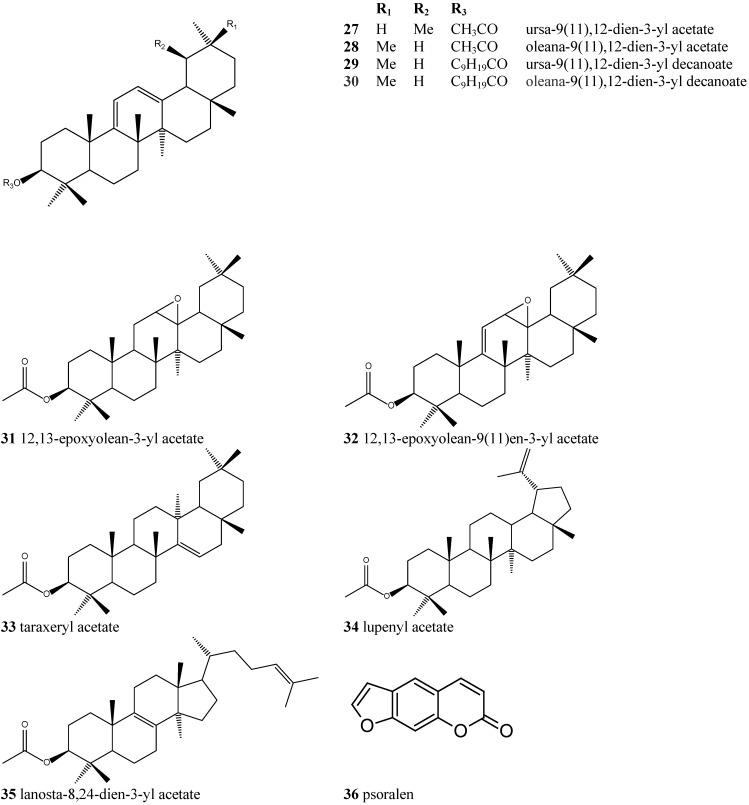
Chemical structures of the other compounds detected by GC-MS in *Dorstenia* *arifolia*.

The chemical constituents of the genus *Dorstenia* have been reported to be coumarins/ furanocoumarins, flavonoids, triterpenoids and triterpenoid esters [15]. This is the first time that long-chain alkanoic acid serial esters (Figure 1 and Figure 2) have been isolated from the *Dorstenia* genus and identified as isomeric pairs.

Hydrolysis of **7**–**12** and of **23**–**26** yielded **1**–**2** and **17**–**18**, respectively, dodecanoic acid and decanoic acid (Section 3). The structures of dodecanoic acid and decanoic acid were confirmed by GC-MS in the form of their methyl esters. Tetradecanoic acid was not detected. For the other compounds hydrolyses were not performed.

Most of the triterpenes found belong to the oleanene/ursene series, characterized by a base peak at *m/z* 218. Unequivocal differentiation between α- and β-amyrin (**1**, **2**) could be seen by examination of the relative intensities of the peaks at *m/z* 189 and 203: β-amyrin (**2**) has a *m/z* 203 peak around twice the intensity of the *m/z* 189 peak, while α-amyrin (**1**) spectra shows both peaks with similar intensities. The triterpenes of the 11-oxo-α-amyrin (**17**) and 11-oxo-β-amyrin (**18**) types present as characteristic signals *m/z* 232, *m/z* 273 and *m/z* 135, the latter being quite abundant. Taraxeryl acetate (**33**) was identified only by MS, mainly due to the base peak at *m/z* 204, which originates from rings D and E of an D^14^-taraxerene. Another important peak is at *m/z* 344, which originates from a retro Diels-Alder decomposition with ring-D opening and confirms both the unsaturation and the presence of an acetoxy group at C-3. The most important feature at glutinyl acetate (**16**) being the base peak at *m/z* 274, followed by a peak at *m/z* 259 (274-Me), which characterizes D^5^-unsaturated skeletal (Table 1) [16].

The presence of a Δ^12^-double bond was corroborated by signals at δ 145.2 and 121.6; 139.5 and 124.3 ppm in the ^13^C-NMR spectrum, assigned to C-12 and C-13, respectively, of olean-12-ene-type and urs-12-en-type skeletons (**27**, **28**, **29**, **30**). Analysis of the mixtures assigned the signal around 170 ppm to the carboxylic group of fatty esters [16].

**Table 1:** Relevant MS data of the compounds identified from *Dorstenia* *arifolia* (EI, 70 eV).

Compound	Fragments, *m/z* (relative abundance)
1	C_30_H_50_O: 426 (M^+^, 11), 218 (100), 203 (22), 189 (22)
2	C_30_H_50_O: 426 (M^+^, 5), 218 (100), 203 (44), 189 (17)
3	C_32_H_52_O_2_: 468 (M^+^, 11), 408 ([M-HAc], 5), 218 (100), 203 (22), 189 (28)
4	C_32_H_52_O_2_: 468 (M^+^, 5), 218 (100), 203 (44), 189 (17)
5 *	C_38_H_24_O_2_: 552 (M^+^, 5), 218 (100), 203 (22), 189 (33)
6 *	C_38_H_24_O_2_: 218 (100), 203 (56), 189 (28)
7	C_40_H_68_O_2_: 580 (M^+^, 4), 409 ([M-HDec + H], 3), 218 (100), 203 (19), 189 (14)
8	C_40_H_68_O_2_: 580 (M^+^, 2), 409 ([M-HDec + H], 1), 218 (100), 203 (30), 189 (14)
9	C_42_H_72_O_2_: 608 (M^+^, 4), 409 ([M-HDod + H], 3), 218 (100), 203 (13), 189 (17)
10	C_42_H_72_O_2_: 608 (M^+^, 2), 409 ([M-HDod + H], 2), 218 (100), 203 (28), 189 (13)
11	C_44_H_76_O_2_: 636 (M^+^, 3), 409 ([M-HTet + H], 3), 218 (100), 203 (12), 189 (16)
12	C_44_H_76_O_2_: 636 (M^+^, 1), 409 ([M-HTet + H], 2), 218 (100), 203 (25), 189 (12)
13 *	C_46_H_80_O_2_: 664 (M^+^, 5), 409 ([M-HHex + H], 5), 218 (100), 203 (11), 189 (17)
14 *	C_46_H_80_O_2_: 409 ([M-Hex + H], 5), 218 (100), 203 (33), 189 (17)
15	C_30_H_50_O: 426 (M^+^, 5), 408 (5), 259 (100), 274 (83)
16	C_32_H_52_O_2_: 468 (M^+^, 11), 259 (100), 274 (94), 408 ([M-HAc], 5)
17	C_30_H_48_O_2_: 440 (M^+^, 22), 408 (5), 273 (89), 232 (78), 135 (100)
18	C_30_H_48_O_2_: 440 (M^+^, 11), 408 (5), 273 (100), 232 (44), 135 (67)
19	C_32_H_50_O_3_: 482 (M^+^, 5), 407 ([M-HAc + H], 5), 232 (61), 273 (61), 135 (100)
20	C_32_H_50_O_3_: 482 (M^+^, 5), 407 ([M-HAc + H], 5), 232 (55), 273 (94), 135 (100)
21	C_38_H_62_O_3_: 566 (M^+^, 5), 423 (5), 407 ([M-HOct + H], 11), 232 (83), 273 (83), 135 (100)
22	C_38_H_62_O_3_: 566 (M^+^, 5), 423 (5), 407 ([M-HOct + H], 5), 232 (55), 273 (100), 135 (72)
23	C_40_H_66_O_3_: 594 (M^+^, 5), 423 (11), 407 ([M-HDec + H], 11), 232 (89), 273 (89), 135 (100)
24	C_40_H_66_O_3_: 594 (M^+^, 5), 423 (5), 407 ([M-HDec + H], 5), 232 (50), 273 (100), 135 (67)
25	C_42_H_70_O_3_: 622 (M^+^, 5), 407 ([M-HDod + H], 11), 232 (100), 273 (94)
26	C_42_H_70_O_3_: 622 (M^+^, 5), 407 ([M-HDod + H], 15), 232 (55), 273 (100)
27 *	C_32_H_50_O_2_: 466 (M^+^, 100), 451 (5), 407 ([M-HAc + H], 5), 255 (50)
28 *	C_32_H_50_O_2_: 466 (M^+^, 100), 451 (11), 407 ([M-HAc + H], 5), 255 (50)
29 *	C_40_H_66_O_2_: 578 (M^+^, 100), 563 (5), 407 ([M-HDec + H], 5), 391 (22), 255 (50)
30 *	C_40_H_66_O_2_: 578 (M^+^, 100), 563 (22), 407 ([M-HDec + H], 11), 391 (28), 255 (28)
31	C_32_H_52_O_3_: 484 (M^+^, 17), 466 (11), 234 (100)
32 *	C_32_H_50_O_3_: 482 (M^+^, 17), 466 (11), 234 (100)
33 *	C_32_H_52_O_2_: 468 (M^+^, 5), 453 (11), 393 (11), 344(39), 269 (33), 204 (100)
34 *	C_32_H_52_O_2_: 468 (M^+^, 11), 408 (11), 204 (11), 189 (100)
35 *	C_32_H_52_O_2_: 468 (M^+^, 11), 453 (39), 393 (56), 353 (11)
36	C_11_H_6_O_3_: 186 (M^+^, 100), 158 (94), 130 (33), 102 (56)

HAc: acetic acid; HOct: octanoic acid; HDec: decanoic acid; HDod: dodecanoic acid; HTet: tetradecanoic acid; HHex: hexadecanoic acid. * Compounds absent in hexane extract of leaves of *D.* *arifolia* (*Da*EHF).

Nine minor triterpenes with different skeletons were also identified in *Dorstenia arifolia* besides a furanocoumarin (**36**) (Figure 2). These different triterpenes were analysed by GC-MS. Table 2 shows the ^13^C-NMR chemical shifts characteristic for the major triterpenes found in *D. arifolia*.

**Table 2:** ^13^C-NMR data for α-amyrin (α-Am), β-amyrin (β-Am), 11-oxo-α-amyrin (11-oxo-α) and 11-oxo-β-amyrin (11-oxo-β) identified from *Dorstenia* *arifolia* [100 MHz, δ (ppm), CDCl_3_].

Carbon	α-Am	β-Am	11-oxo-α	11-oxo-β
3	79.0	79.0	78.8	78.8
11	23.6	23.6	199.8	200.3
12	124.4	121.8	130.4	128.1
13	139.5	145.2	164.9	170.6

In addition to the peaks assigned to the major characteristic fragments of each triterpenoid skeleton, the mass spectrum revealed the molecular ion peaks corresponding to the triterpene esters in the mixtures. The equation below allowed us to find the number of units of each CH_2_ acyl unit. Thus, for α-amyrin hexadecanoate (**13**), for example: M^+^ − 664 [15 (CH3) + 44 (O=C–O) + 409] = 14n.

## 3. Experimental

### 3.1. General Procedures

^1^H-NMR (400 MHz) and ^13^C-NMR (100 MHz) experiments were carried out on a Varian (mod. 400/54/ASP) instrument; chemical shifts were recorded in δ (ppm) to TMS. GC/MS data were obtained on a Shimadzu QP5000 unit. Column chromatography was carried using silica gel 60 (Akros 0.04–0.073 mm), and silica gel TLC plates employing ceric sulfate spray reagent and UV light (254/365 nm) to monitor chromatographic profiles.

### 3.2. Plant Material

Samples of *D. arifolia* Lam. (Moraceae) were collected in Rio de Janeiro, Brazil. The botanical identification was provided by Dr. Marcelo Dias Machado Vianna Filho and a voucher specimen (RB 517081) was deposited in the Herbarium of the Jardim Botânico do Rio de Janeiro, Rio de Janeiro, Brazil.

### 3.3. Extraction and Isolation

Plant material was dried at 40 °C, with forced ventilation, before being powdered. The terpenoidalfractions which contained mainly triterpene esters were obtained by silica gel open-column liquid chromatography (PLC) of the crude plant extracts and selected by GC-MS and NMR (^1^H- or ^13^C-) analysis of the mixture. Powdered leaves (10 g) of *D. arifolia* were successively extracted with *n*-hexane. The solvent was removed under reduced pressure to yield the hexane crude extract (*Da*EHF). *Da*EHF was chromatographed on silica gel (0.063–0.200 mm, Merck, Darmstadt, Germany) using hexane-ethyl acetate of increasing polarity, which yielded fractions in mixture: A, B, C, D, E and F. Fraction A (1.6 g) was submitted to a silica gel column chromatography (*Da*EHF) eluted with hexane:ethyl acetate 3% to yield the mixture (Figure 1): β-amyrin decanoate (**8**), α-amyrin decanoate (**7**), β-amyrin dodecanoate (**10**), α-amyrin dodecanoate (**9**), β-amyrin tetradecanoate (**12**), α-amyrin tetradecanoate (**11**), 11-oxo-α-amyrin decanoate (**23**), 11-oxo-β-amyrin decanoate (**24**), 11-oxo-α-amyrin dodecanoate (**25**) and 11-oxo-β-amyrin dodecanoate (**26**).

Fraction B (2.7 g) eluted on a silica gel column chromatography (*Da*EHF) was submitted to column chromatography using Sephadex LH-20 and CHCl_3_/MeOH (7:3) as eluent to yield (Figure 1): β-amyrin acetate (**4**), α-amyrin acetate (**3**) and glutinyl acetate (**16**).

Fraction C (55 mg) was eluted on a silica gel column chromatography (*Da*EHF) with hexane/ethyl acetate 3% and submitted to new silica gel column chromatography to yield (Figure 1): 11-oxo-α-amyrin octanoate (**21**), 11-oxo-β-amyrin octanoate (**22**), 11-oxo-α-amyrin decanoate (**23**), 11-oxo-β-amyrin decanoate (**24**).

Fractions D (796 mg) and E (287 mg) were eluted on a silica gel column chromatography with hexane/ethyl acetate 3% and submitted to a new silica gel column chromatography to yield (Figure 1): α-amyrin (**1**).

Fraction F was eluted on a silica gel column chromatography (*Da*EHF) with hexane/ethyl acetate 10% and submitted to a new silica gel column chromatography to yield (Figure 1 and Figure 2): ursa-9(11),12-dien-3-yl acetate (**27**), olean-9(11),12-dien-3-yl acetate (**28**), 12,13-epoxyolean-9(11)en-3-yl acetate (**32**), 11-oxo-α-amyrin acetate (**19**) and 11-oxo-β-amyrin acetate (**20**).

Powdered rhizomes (10 g) of *D. arifolia* were exhaustively extracted with *n*-hexane. The solvent was removed under reduced pressure to yield the hexane extract (*Da*EHR). *Da*EHR was chromatographed over silica gel (0.063–0.200 mm, Merck) using hexane-ethyl acetate of increasing polarity, which yielded mixed fractions G, H, I, J, K, L, M and N.

Fraction G (1 g) was eluted on a silica gel column chromatography with hexane/ethyl acetate 1% to yield the mixture (Figure 1): β-amyrin decanoate (**8**), α-amyrin decanoate (**7**), β-amyrin dodecanoate (**10**), α-amyrin dodecanoate (**9**), β-amyrin tetradecanoate (**12**) and α-amyrin tetradecanoate (**11**).

Fraction H (1 g) was eluted on a silica gel column chromatography with hexane/ethyl acetate 1% to yield (Figure 1): 11-oxo-α-amyrin decanoate (**23**), β-amyrin hexadecanoate (**14**), α-amyrin hexadecanoate (**13**) beyond the constituents from Fraction H.

Fraction I (339 mg) was eluted on a silica gel column chromatography with hexane/ethyl acetate 1% to yield the pairs of isomers (Figure 1): β-amyrin acetate (**4**), glutinyl acetate (**16**), α-amyrin acetate (**3**), β-amyrin octanoate (**6**), α-amyrin octanoate (**5**), β-amyrin decanoate (**8**), α-amyrin decanoate (**7**), β-amyrin dodecanoate (**10**) and α-amyrin dodecanoate (**9**).

Fraction J (2 g) was eluted on a silica gel column chromatography with hexane/ethyl acetate 1% to yield (Figure 1 and Figure 2): β-amyrin acetate (**4**), glutinyl acetate (**16**), α-amyrin acetate (**3**), β-amyrin decanoate (**8**), 11-oxo-α-amyrin acetate (**19**), α-amiryn decanoate (**7**), β-amyrin octanoate (**6**), α-amyrin octanoate (**5**), β-amyrin dodecanoate (**10**), 11-oxo-α-amyrin decanoate (**23**), α-amyrin dodecanoate (**9**) and esters of different triterpene skeletons: ursa-9(11),12-dien-3-yl acetate (**27**), oleana-9(11),12-dien-3-yl acetate (**28**), ursa-9(11),12-dien-3-yl decanoate (**29**), oleana-9(11),12-dien-3-yl decanoate (**30**), 12,13-epoxiolean-3-yl acetate (**31**), 12,13-epoxyolean-9(11)-en-3-yl acetate (**32**), taraxeryl acetate (**33**), lupenyl acetate (**34**) and lanosta-8,24-dien-3-yl acetate (**35**).

Fraction K (280 mg) was eluted on a silica gel column chromatography with hexane/ethyl acetate 2% to yield (Figure 1 and Figure 2): ursa-9(11),12-dien-3-yl acetate (**27**), olean-9(11),12-dien-3-yl acetate (**28**), α- amyrin acetate (**3**), glutinol (**15**), 11-oxo-β-amiryn octanoate (**22**), 11-oxo-α-amyrin decanoate (**23**), 11-oxo-β-amyrin decanoate (**24**) and 11-oxo-β-amyrin dodecanoate (**26**).

Fraction L (36 mg) was eluted on a silica gel column chromatography with hexane/ethyl acetate 2% to yield the pair of isomers (Figure 2) ursa-9(11),12-dien-3-yl acetate (**27**) and oleana-9(11),12-dien-3-yl acetate (**28**).

Fraction M (922 mg) was eluted through a silica gel column chromatography with hexane/ethyl acetate 3% to yield triterpenoid skeletons (Figure 1) without esterification: α-amyrin (**1**) and β- amyrin (**2**) and the esters of triterpenes 11-oxo-α-amyrin acetate (**19**) and 11-oxo-β-amyrin acetate (**20**).

Fraction N (135 mg) was eluted on a silica gel column chromatography with hexane/ethyl acetate 15% and was submitted to a small column chromatography using Sephadex LH-20 and CHCl_3_/MeOH (1:1) as eluent to yield a furanocoumarin (1 mg) (Figure 2).

All compounds were identified by interpretation of the results of the spectra and comparison with literature data.

### 3.4. Chromatographic Analysis

GC-MS analysis was performed by using a GC-MS QP5000 Shimadzu, with electron impact ionization (70 eV). The column used was a DB-5MS (30 m × 0.25 mm × 0.25 µm) with injector temperature at 290 °C and GC-MS interface temperature at 250 °C. Column temperature was programmed from 100 °C at 320 °C (held during 120 min), ranging 10 °C/min. Helio was used as carrier gas. The mixtures A-N were analyzed by GC-MS which furnished a fast differentiation among important skeletons. The NMR data were only used to confirm the results proposed by mass spectra.

### 3.5. Basic Hydrolysis of Triterpene Ester Derivatives

Some triterpene ester derivatives (compounds **7**–**12** and **22**–**26**) were submitted to hydrolysis by adding 4 mL of a solution of NaOH in MeOH 0.5 N to 100 mg of mixture for 10 hours. After this time, the reaction medium was saturated with NaCl 360 g/L and the triterpenes were extracted with CHCl_3_. The aqueous solution was acidified with 4 mL of HCl 0.5 N followed by the extraction with CHCl_3_. This resultant organic phase was washed and dried over Na_2_SO_4_, yielding the fatty acids.

## 4. Conclusions

GC-MS has proved to be a very powerful tool affording both the separation and the individual characterization of terpenoidal isomers which could not to be separated by conventional PLC procedures. MS data furnish a fast differentiation among important skeleton types, some of them with potential biological interest shown in literature. 

Pentacyclic triterpenes and a furocoumarin from *Dostenia arifolia* were identified. These compounds may be related to the folk utilization of *Dorstenia* species as antiophidicals. The utilization of *Dorstenia* plants as antiophidicals may be inferred to be both due to a venom-inactivating action and to the analgesic and antiinflamatory properties of the various triterpenes [11].

## References

[1] Abegaz B.M., Ngadjui B.T., Dongo E., Ngameni B., Nindi M.N., Bezabih M. (2002). Chalcones and other constituents of *Dorstenia prorepens* and *Dorstenia zenkeri*. Phytochemistry.

[2] Ngadjui B.T., Dongo B.M., Fotso S., Tamboue H. (2002). Dinklagins A, B and C: Three prenylated flavonoids and other constituents from the twigs of *Dorstenia dinklagei*. Phytochemistry.

[3] Omisore N.O.A., Adewunmi C.O., Iwalewa E.O., Ngadjui B.T., Watchueng J., Abegaz B.M., Ojewole J.A.O. (2004). Antinociceptive and anti-inflammatory effects of *Dorstenia barteri* (Moraceae) leaf and twig extracts in mice. J. Ethnopharmacol..

[4] Ngameni B., Touaibia M., Patnam R., Belkaid A., Sonna P., Ngadjui T., Annabi B., Roy R. (2006). Inhibition of MMP-2 secretion from brain tumor cells suggests chemopreventive properties of a furanocoumarin glycoside and of chalcones isolated from the twigs of *Dorstenia turbinate*. Phytochemistry.

[5] Vouffo B., Krohn K., Kouam S.F., Hussain H., Dongo E., Meier K., Schulz B. (2008). Dinklagenonoate: A new isobauerane-type triterpenoid and other minor constituents from the twigs of *Dorstenia dinklagei*. Biochem. Syst. Ecol..

[6] Tabopda T.K., Ngoupayo J., Awoussong P.K., Mitaine-Offer A.C., Ali M.S., Ngadjui B.T., Lacaille-Dubois M.A. (2008). Triprenylated flavonoids from *Dorstenia psilurus* and their R-glucosidase inhibition properties. J. Nat. Prod..

[7] Heinke R., Franke K., Michels K., Wessjohann L., Ali N.A.A., Schmidt J. (2012). Analysis of furanocoumarins from Yemenite *Dorstenia* species by liquid chromatography/electrospray tandem mass spectrometry. J. Mass. Spectrom..

[8] Ngadjui B.T., Abegaz B.M. (2003). The chemistry and pharmacology of the genus *Dorstenia* (Moraceae). J. Nat. Prod..

[9] Silva M.L., David J.P., Silva L.C.R.C., Santos R.A.F., David J.M., Lima L.S., Pedro S.R., Fontana R. (2012). Bioactive oleanane, oupane and ursane triterpene acid derivatives. Molecules.

[10] Hernández-Vázquez L., Mangas S., Palazón J., Navarro-Ocaña A. (2010). Valuable medicinal plants and resins: Commercial phytochemicals with bioactive properties. Ind. Crop. Prod..

[11] Vilegas J.H.Y., Lanças F.M., Vilegas W., Pozetti G.L. (1997). Further triterpenes, steroids and furocoumarins from brazilian medicinal plants of *Dorstenia* genus (Moraceae). J. Braz. Chem. Soc..

[12] Barreiros M.L., David J.M., Queiroz L.P., David J.P. (2005). Flavonoids and triterpenes from leaves of *Erythroxylum nummularia*. Biochem. Syst. Ecol..

[13] Mendes C.C., Cruz F.G., David J.M., Nascimento I.P., David J.P. (1999). Triterpenos esterificados com ácidos graxos e ácidos triterpênicos isolados de *Byrosonima microphylla*. Quim. Nova.

[14] Zapata-Sudo G., Mendes C.F., Kartnaller M.A., Fortes TO., Freitas N.F.B., Kaplan M.A.C., Sudo R.T. (2010). Sedative and anticonvulsant activities of methanol extract of *Dorstenia arifolia* in mice. J. Ethnopharmacol..

[15] Poumale H.M.P., Awoussonga K.P., Randrianasoloc R., Christophe C.F.S., Ngadjuia B.T., Shiono Y. (2012). Long-chain alkanoic acid esters of lupeol from *Dorstenia harmsiana* Engl. (Moraceae). Nat. Prod. Res..

[16] Fingolo C.E. (2012). Phytochemical Strategies for the Biological Diversity Sustainable Use. Ph.D. Thesis.

